# Brillouin Frequency Shift Extraction Based on AdaBoost Algorithm

**DOI:** 10.3390/s22093354

**Published:** 2022-04-27

**Authors:** Huan Zheng, Feng Xiao, Shijie Sun, Yali Qin

**Affiliations:** Institute of Fiber-Optic Communication and Information Engineering, College of Information Engineering, Zhejiang University of Technology, Hangzhou 310023, China; yhs954368007@163.com (F.X.); 2112003151@zjut.edu.cn (S.S.)

**Keywords:** distributed fiber sensing, Brillouin scattering, Brillouin frequency shift, AdaBoost algorithm

## Abstract

The Brillouin Optical Time-Domain Analyzer assisted by the AdaBoost Algorithm for Brillouin frequency shift (BFS) extraction is proposed and experimentally demonstrated. The Brillouin gain spectrum classification under different BFS is realized by iteratively updating the weak classifier in the form of a decision tree, forming several base classifiers and combining them into a strong classifier. Based on the pseudo-Voigt curve training set with noise, the performance of the AdaBoost Algorithm is studied, and the influence of different signal-to-noise ratio (SNR), frequency range, and frequency step is also studied. Results show that the performance of BFS extraction decreases with the decrease in SNR, the reduction in frequency range, and the increase in frequency step.

## 1. Introduction

The advantages of distributed optical fiber sensors include distributed sensing, high spatial resolution, large dynamic range, real-time monitoring, etc. The technique of distributed fiber sensing can be widely applied in various areas, such as industrial infrastructure health monitoring, long-haul vibration detection, and quick fault location [1,2,3]. The Brillouin Optical Time-Domain Analyzer (BOTDA), as one of many distributed optical fiber sensors, can be used for monitoring both temperature and strain in ultra-long sensing ranges [4]. In order to obtain temperature and strain information from BOTDA, Brillouin frequency shift (BFS) needs to be extracted from the measured Brillouin Gain Spectrum (BGS).

One method for BFS extraction is curve fitting [5,6,7,8]. The curves can be fitted by using Lorentzian curve, parabolic curve, pseudo-Voigt curve, etc. [5,6]. However, the curve fitting method is highly dependent on the initial conditions. As for the BGS with low signal-to-noise ratio (SNR), the initial parameters need to be adjusted carefully and often lead to fitting failures [7]. In addition, the curve fitting method requires a small frequency step, because a large frequency step could reduce the number of fitting points and affect the fitting performance [8].

In order to avoid the problem of initial parameter adjustment in the curve fitting method, the Cross-Correlation Method (XCM) is proposed to extract BFS [8,9]. XCM does not have the problem of initial parameter setting, but it requires data interpolation processing to up-sample the measured BGS, which could be time consuming. As a result, there is a trade-off between accuracy and processing speed [9].

The method of Artificial Neural Network (ANN) has also been introduced in the BOTDA system [10,11,12]. ANN is proved to be an effective method for BFS extraction; however, the number of intermediate layers and the number of neurons in layers need to be designed carefully [10]. Moreover, the training process is always complicated and time consuming. The method can be converted to the local optimal solution, which limits its applications [11,12].

In recent years, Support Vector Machine (SVM) has also been proposed to extract temperature from the whole BGS [13,14,15]. However, because SVM is essentially a binary classifier, it is often necessary to construct hundreds of support vectors to extract temperature information. The error of support vectors could also affect the accuracy of extraction.

Here, we propose a novel method for BFS extraction. The method is based on the AdaBoost Algorithm. The basic idea of AdaBoost is to train different weak classifiers with the same training sets and combine them to build a strong classifier. The advantages of the AdaBoost Algorithm include freedom of extraction failure and simplicity of parameter adjustments [16,17]. Generally, the decision tree or neural network can be chosen as the weak classifier [18,19]. In this study, the BFS extraction is treated as a supervised classification problem. The algorithm of the AdaBoost method is applied to extract BFS from BGS.

The article is organized as follows: In Section II the principle of the AdaBoost Algorithm is introduced, and the AdaBoost Algorithm for extracting BFS is illustrated. In Section III, the performance of BFS extraction by AdaBoost is numerically studied under different SNR, frequency range, and frequency step. In Section IV, the BFS extraction by the AdaBoost Algorithm is experimentally studied. The result of the experiment is compared with that of the simulation. Finally, the study is briefly concluded in Section V.

## 2. Theoretical Model

### 2.1. Principle of AdaBoost

AdaBoost is a supervised learning algorithm for solving binary classification problems. The principle of AdaBoost is shown in the details below.

The decision tree is chosen to be the weak classifier. The algorithm of Classification And Regression Tree (CART) is introduced to train the decision tree. For instance, *X_n_*_×*m*_ stands for the properties of the training set, which can be expressed as follows [20]:

(1) $$X_{n\times m}=\left[\begin{array}{cccc} X_{1,1} & X_{1,2} & \cdots & X_{1,m}\\ X_{2,1} & X_{2,2} & \cdots & X_{2,m}\\ \vdots & \vdots & & \vdots \\ X_{n,1} & X_{n,2} & \cdots & X_{n,m} \end{array}\right]=\left[\begin{array}{c} {\overset{🠒}{X}}_{1}\\ {\overset{🠒}{X}}_{2}\\ \vdots \\ {\overset{🠒}{X}}_{n} \end{array}\right]$$

(2) $$Y_{n\times 1}={\left[\begin{array}{cccc} y_{1} & y_{2} & \cdots & y_{n} \end{array}\right]}^{T}$$

**Figure 1:**
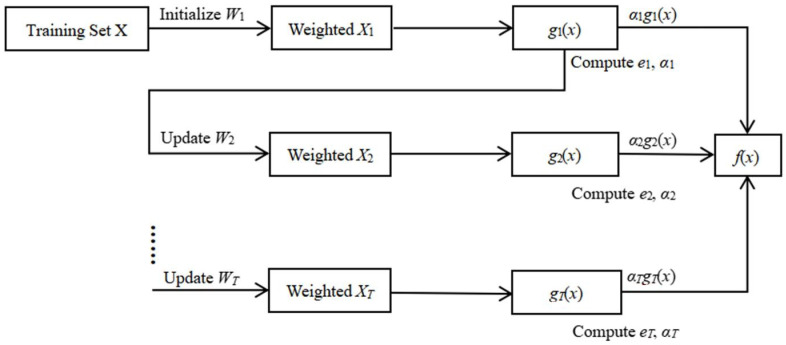
The principle of AdaBoost Algorithm, where *X*, *W_i_*, *X_i_*, *e_i_*, *α_i_*, *g_i_*(*x*), and *f*(*x*) represent the labeled training samples, the weight set, the weighted sample set, the classification error, the weak classifier coefficient, the weak classifier, and the final strong classifier, respectively.

*Y_n_*_×1_ represents for the labels of one sample in the training set, where *y_i_* = 0 or 1. After the training process, a tree structure is constructed, which can be denoted as a model of $\overset{🠒}{p}$; then, the decision tree can be expressed as follows [21]:

(3) $$Y^{\prime }=g(X,\overset{🠒}{p})$$

where *Y′* represents the output of the decision tree. The main process of AdaBoost is shown in Figure 1.

In the algorithm of AdaBoost, a weight factor *w_i_* is introduced for each sample in the training set, where *i* = 1, 2..., *n*, standing for the index of that sample. *W_n_*_×1_ is defined as the initial sample weight [20]:

(4) $$W_{n\times 1}={\left[\begin{array}{cccc} w_{1} & w_{2} & \cdots & w_{n} \end{array}\right]}^{T}$$

Here, an operation ⊗ is defined as follows [22]:

(5) $$W_{n\times 1}⊗X_{n\times m}=\left[\begin{array}{cccc} w_{1}x_{1,1} & w_{1}x_{1,2} & \cdots & w_{1}x_{1,m}\\ w_{2}x_{2,1} & w_{2}x_{2,2} & \cdots & w_{2}x_{2,m}\\ \vdots & \vdots & & \vdots \\ w_{n}x_{n,1} & w_{n}x_{n,2} & \cdots & w_{n}x_{n,m} \end{array}\right]$$

During iteration, the weak classifier is trained based on training samples, and then the weight of each sample is updated according to the error rate and classification error of this particular sample. The initial weight of the sample *W*_1_ is set to [22]

(6) $$W_{n\times 1}^{1}={\left[\begin{array}{cccc} \frac{1}{n} & \frac{1}{n} & \cdots & \frac{1}{n} \end{array}\right]}^{T}$$

The characteristics of training set *X*_1_ used for the weak classifier are

(7) $$X_{1}=W_{n\times 1}^{1}⊗X_{n\times m}$$

Furthermore, the output $Y_{n\times 1}^{1}$ is

(8) $$Y_{n\times 1}^{1}=g_{1}(X_{1},\overset{🠒}{p})$$

The error rate *e*_1_ can be obtained by

(9) $$e_{1}=P(g_{1}(X_{1},\overset{🠒}{p})\neq Y_{n\times 1})$$

According to the calculated error rate, the weight of weak classifier *a*_1_ is obtained by

(10) $$a_{1}=\frac{1}{2}\log\frac{1-e_{1}}{e_{1}}$$

After that, the weight of sample $w_{i}^{2}$ can be updated by

(11) $$w_{i}^{2}=\frac{w_{i}^{1}}{Z_{1}}\exp(-a_{1}y_{i}g_{1}({\overset{🠒}{x}}_{i},\overset{🠒}{p})),i=1,2,\ldots ,n$$

(12) $$Z_{1}=\sum_{t=1}^{n}w_{t}^{1}\exp(-a_{1}y_{t}g_{1}({\overset{🠒}{x}}_{t},\overset{🠒}{p}))$$

The classification error of the weak classifier generated by AdaBoost will be gradually decreased after iterations of *T* steps.

AdaBoost combines all the weak classifiers to construct a strong classifier, in which the weak classifier with the lower classification error will obtain more weight.

It is worth noting that the resulting training error drops exponentially and rapidly to zero when the classification error of the weak classifier reaches only a little less than 50%. Therefore, the weak classifier needs to be slightly better than the random classifier.

The whole algorithm of AdaBoost is shown in detail (Algorithm 1), as follows [22].
**Algorithm 1:** AdaBoost for supervised classification.Give the number of iterations *T* and input the labeled training sample set *F*, *k*←0**repeat**According to the weighted training samples, the weak classifier $g_{k}(X_{k},\overset{🠒}{p})$ is trained.Calculate error rate $e_{i}=P(g_{i}({\bar{X}}_{i},\overset{🠒}{p})\neq Y_{i}),i=1,2,\ldots ,n$Compute coefficients $a_{k}=\frac{1}{2}\log\frac{1-e_{k}}{e_{k}}$Compute $Z_{k}=\sum_{t=1}^{n}\omega_{t}^{k}\exp(-a_{k}y_{t}g_{k}({\bar{X}}_{t},\overset{🠒}{p}))$Update $w_{i}^{k+1}=\frac{w_{i}^{k}}{Z_{k}}\exp(-a_{k}y_{i}g_{k}({\bar{X}}_{i},\overset{🠒}{p})),i=1,2,\ldots ,n$**Until** *k* < T, *k*←*k* + 1

### 2.2. Principle of BFS Extraction by AdaBoost

In order to solve multiple classification problem of BFS extraction, for a given sample, firstly two different labels are chosen from the training set, and the AdaBoost Algorithm is applied for this exact binary classification problem. Then, this routine is repeated until all different label pairs in the training set are chosen, and finally the final label for the input sample is decided by voting.

In this study, the pseudo-Voigt curve is selected to make it applicable to conditions of different pump pulse widths, and $c=\left(\alpha ,p,\Delta v\right)$ is defined as a combination vector of Lorentz ratio *α*, curve gain parameter *p* and curve line width Δv. Assume that the value range of center frequency *v*_0_ is *v*_0_ = [*v*_01_, *v*_02_…, *v*_0*n*_] and the value range of *c* is *c* = [*c*_1_, *c*_2_…, *c_m_*]. The scanning frequency range of curve is *v_i_* = [*v_i_*_1_, *v_i_*_2_…, *v_ik_*]. The Brillouin gain set *F_mn_*_×*k*_ can be expressed as follows:

(13) $$F_{mn\times k}=\left[\begin{array}{cccc} S(v_{01},c_{1},v_{i1}) & S(v_{01},c_{1},v_{i2}) & \cdots & S(v_{01},c_{1},v_{ik})\\ S(v_{01},c_{2},v_{i1}) & S(v_{01},c_{2},v_{i2}) & \cdots & S(v_{01},c_{2},v_{ik})\\ \vdots & \vdots & \; & \vdots \\ S(v_{01},c_{m},v_{i1}) & S(v_{01},c_{m},v_{i2}) & \cdots & S(v_{01},c_{m},v_{ik})\\ S(v_{02},c_{1},v_{i1}) & S(v_{02},c_{2},v_{i2}) & \cdots & S(v_{02},c_{2},v_{ik})\\ S(v_{02},c_{2},v_{i1}) & S(v_{02},c_{2},v_{i2}) & \cdots & S(v_{02},c_{2},v_{ik})\\ \vdots & \vdots & \; & \vdots \\ S(v_{02},c_{m},v_{i1}) & S(v_{02},c_{m},v_{i2}) & \cdots & S(v_{02},c_{m},v_{ik})\\ \vdots & \vdots & \; & \vdots \\ S(v_{0n},c_{1},v_{i1}) & S(v_{0n},c_{1},v_{i2}) & \cdots & S(v_{0n},c_{1},v_{ik})\\ S(v_{0n},c_{2},v_{i1}) & S(v_{0n},c_{2},v_{i2}) & \cdots & S(v_{0n},c_{2},v_{ik})\\ \vdots & \vdots & \; & \vdots \\ S(v_{0n},c_{m},v_{i1}) & S(v_{0n},c_{m},v_{i2}) & \cdots & S(v_{0n},c_{m},v_{ik}) \end{array}\right]$$

Here, the Brillouin gain subsets *F_i_* and *F_j_* from $F_{mn\times k}$ are chosen to be

(14) $$F_{i}=S_{m\times k}(v_{0i},c,v_{i})=\left[\begin{array}{c} R(v_{0i},c_{1},v_{i})\\ R(v_{0i},c_{2},v_{i})\\ \vdots \\ R(v_{0i},c_{m},v_{i}) \end{array}\right]=\left[\begin{array}{cccc} S(v_{0i},c_{1},v_{i1}) & S(v_{0i},c_{1},v_{i2}) & \cdots & S(v_{0i},c_{1},v_{ik})\\ S(v_{0i},c_{2},v_{i1}) & S(v_{0i},c_{2},v_{i2}) & \cdots & S(v_{0i},c_{2},v_{ik})\\ \vdots & \vdots & & \vdots \\ S(v_{0i},c_{m},v_{i1}) & S(v_{0i},c_{m},v_{i2}) & \cdots & S(v_{0i},c_{m},v_{ik}) \end{array}\right]$$

(15) $$F_{j}=S_{m\times k}(v_{0j},c,v_{i})=\left[\begin{array}{c} R(v_{0j},c_{1},v_{i})\\ R(v_{0j},c_{2},v_{i})\\ \vdots \\ R(v_{0j},c_{m},v_{i}) \end{array}\right]=\left[\begin{array}{cccc} S(v_{0j},c_{1},v_{i1}) & S(v_{0j},c_{1},v_{i2}) & \cdots & S(v_{0j},c_{1},v_{ik})\\ S(v_{0j},c_{2},v_{i1}) & S(v_{0j},c_{2},v_{i2}) & \cdots & S(v_{0j},c_{2},v_{ik})\\ \vdots & \vdots & & \vdots \\ S(v_{0j},c_{m},v_{i1}) & S(v_{0j},c_{m},v_{i2}) & \cdots & S(v_{0j},c_{m},v_{ik}) \end{array}\right]$$

$D_{1}=(\omega_{1,1},\omega_{1,2},\ldots ,\omega_{1,2m});\omega_{1,r}=\frac{1}{2m};r=1,2,\ldots ,2m$ is initialized and the weighted sample set $T_{i,j}^{1}$ is constructed for the binary classification training of the AdaBoost Algorithm [23]:

(16) $$T_{i,j}^{1}=\left[\begin{array}{c} \omega_{1,1}R(f_{i},c_{1},f_{v})\\ \omega_{1,2}R(f_{i},c_{2},f_{v})\\ \vdots \\ \omega_{1,r}R(f_{i},c_{m},f_{v})\\ \omega_{1,r+1}R(f_{j},c_{1},f_{v})\\ \omega_{1,r+2}R(f_{j},c_{2},f_{v})\\ \vdots \\ \omega_{1,2m}R(f_{j},c_{m},f_{v}) \end{array}\right]$$

**Figure 2:**
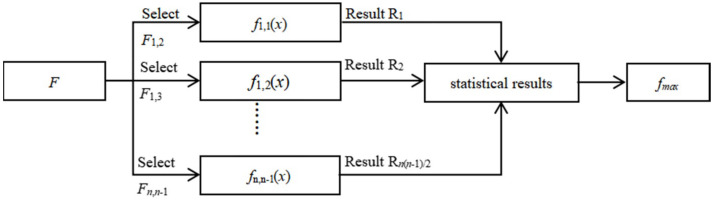
BFS extraction process based on AdaBoost, where *F*, *F_i_*,*_j_*, *f_i_*,*_j_*(*x*), *R_i_,* and *f_max_* are the classifier set, the binary label sample set, the binary classifier, the classification result, and the BFS of output, separately.

For a sample set, the corresponding binary classifier is obtained by $f_{i,j}=sign(\sum_{t=1}^{t_{s}}a_{t}G_{t})$, as shown in Figure 2. Therefore, classifiers can be expressed as:

(17) $$f=[f_{1,2}\;f_{1,3}\;\cdots \;f_{1,n}\;f_{2,3}\;f_{2,4}\;\cdots \;f_{2,n}\;\cdots \;f_{n-1,n}{]}^{T}$$

For the given BGS *S_p_* = *S*(*f_p_*,*c_p_*,*f_vk_*), the classifier set is constructed, the output results of the center frequency of each classifier are counted, and the maximum frequency value *f_max_* is obtained, which should be the extracted BFS corresponding to the given BGS.

## 3. Simulation

The performance of AdaBoost extraction is studied by simulation. The AdaBoost Algorithm, using ideal training samples and training samples with noise, are trained separately.

Here, the ideal training sample can be expressed as [14]:

(18) $$f_{v}=p\frac{a}{1+4{\left(\frac{v_{i}-v_{0}}{\Delta v}\right)}^{2}}+p(1-a)\exp[-4\ln2{\left(\frac{v_{i}-v_{0}}{\Delta v}\right)}^{2}]$$

When the ideal samples are used for training, the Lorentz ratio *α* varies from 0 to 1, with an interval of 0.2. The width of the curves Δv ranges from 10 MHZ to 50 MHz, and the interval is 5 MHz. The curve gain parameter *p* is fixed at 1. The center frequency *v*_0_ ranges from 10,800 MHZ to 10,980 MHz, which is the same as the frequency scanning range with an interval of 1 MHz. Therefore, 6 × 9 × 181 samples can be obtained for model training.

For the training samples with noise, a Gaussian white noise is introduced into the pseudo-Voigt curve. The width of the curves, the curve gain parameter, the center frequency, and the frequency scanning range are the same as that of the ideal training samples. The Gaussian white noise with mean value of 0 and different standard deviations (0.05, 0.10, 0.15, 0.20, 0.25, 0.30, 0.35, 0.40) are added to the training samples, separately. The process with different standard deviations is repeated 5 times, so 6 × 9 × 61 × 5 × 8 samples are finally obtained for model training.

After model training, the model is applied to a test. During the test, the pseudo-Voigt curve with noise is used to simulate BGS, where the line width is fixed at 50 MHz, suggesting a pump signal with the pulse width of around 20 ns. The center frequency is fixed at 10,860 MHz, and the Lorentz ratio is fixed at 1. Here, the SNR is defined as the ratio between the mean amplitude of the Brillouin peak or trace and its standard deviation, which is proportional to the amplitude instead of the power [14].

### 3.1. BGS Extraction under Different SNR

**Figure 3:**
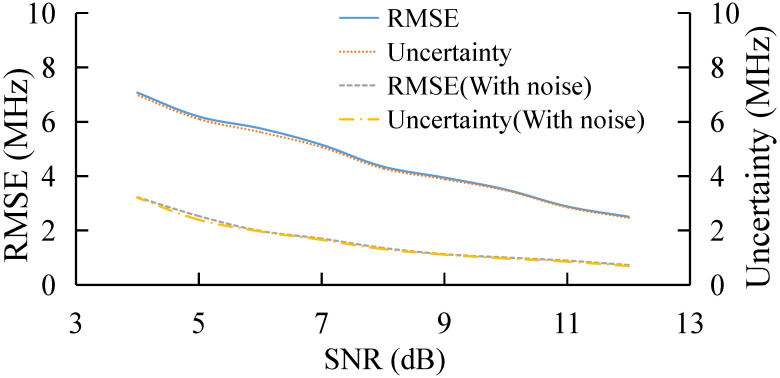
BFS extraction based on training samples with or without noise under different SNR.

The Root Mean Square Error (RMSE) and uncertainty under different SNR based on both training samples, with and without noise, is shown in Figure 3. With the increase in SNR, both the RMSE and uncertainty of the AdaBoost Algorithm decrease, and the values of RMSE and uncertainty are similar under different SNR. It is obvious that the algorithm model obtained by using training samples with noise is better than that without noise. This may be because the pseudo-Voigt curve without noise has only one data sample based on the same feature, while for the pseudo-Voigt curve with noise, there are 40 data samples used for classification training under the same feature.

### 3.2. BGS Extraction under Different Cut-Off Frequency

**Figure 4:**
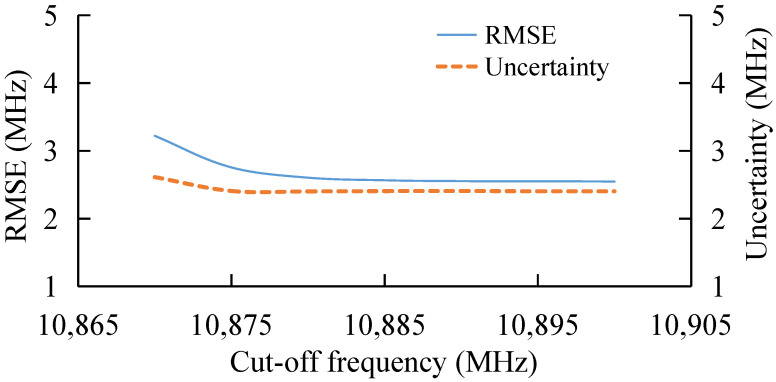
BFS extraction under different cut-off frequency.

Based on the frequency range of 10,800–10,980 MHz, the BGS extraction under different SNR is tested. Here, the initial frequency is fixed at 10,800 MHz and the cut-off frequency is changed. The result is shown in Figure 4. The SNR is fixed at 7 dB.

With the increase in cut-off frequency, both RMSE and uncertainty gradually decrease, and the extraction error tends to be converged when the cut-off frequency is larger than 10,880 MHz, as shown in Figure 4. With the decrease in cut-off frequency, the BGS data set of BOTDA decreases, which leads to the increase in the extracting error.

### 3.3. BGS Extraction under Different Frequency Range

The cut-off frequency is also fixed and the extraction performance under different initial frequencies is investigated. Results show that when the cut-off frequency is fixed at 10,980 MHz and the initial frequency is changed, the extraction error tends to be stable when the initial frequency is less than 10,835 MHz.

**Figure 5:**
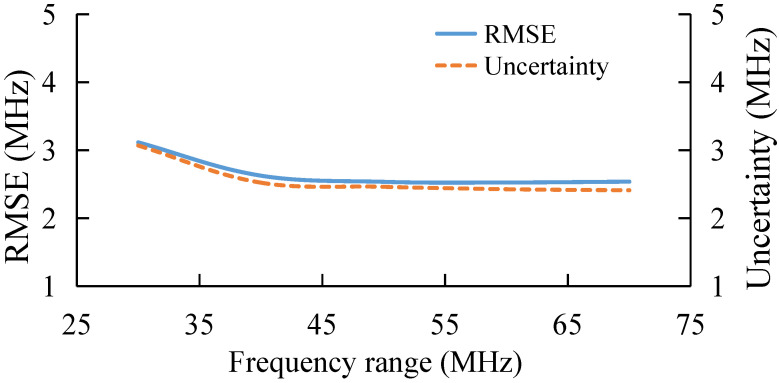
BFS extraction under different frequency range.

Therefore, further analysis is conducted for the frequency range between 10,825 MHz and 10,895 MHz. The results are shown in Figure 5. With the decrease in the frequency range, the BFS extraction accuracy decreases, and the feature number of BGS data increases with frequency range, so the BGS extraction accuracy could be stable when the frequency range is larger than 50 MHz. Overall, AdaBoost can retain extraction accuracy when the frequency range is small.

### 3.4. BGS Extraction under Different Frequency Step

**Figure 6:**
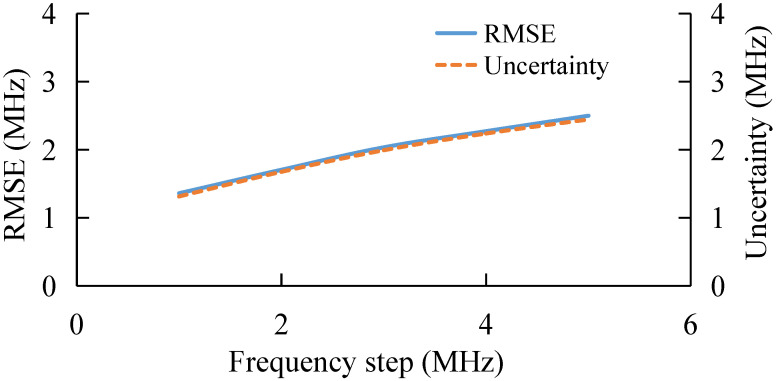
BFS extraction under different frequency step.

The frequency step determines the number of features in each feature vector, i.e., the number of data points on each BGS. Here, the BGS extraction at different frequency step is studied. The results are shown in Figure 6. As the frequency step increases, the extraction performance of AdaBoost gradually deteriorates. Figure 6 shows that with a larger frequency step, the feature set becomes sparser, which could introduce more error into BFS extraction.

## 4. BOTDA Setup and Experiment Results

**Figure 7:**
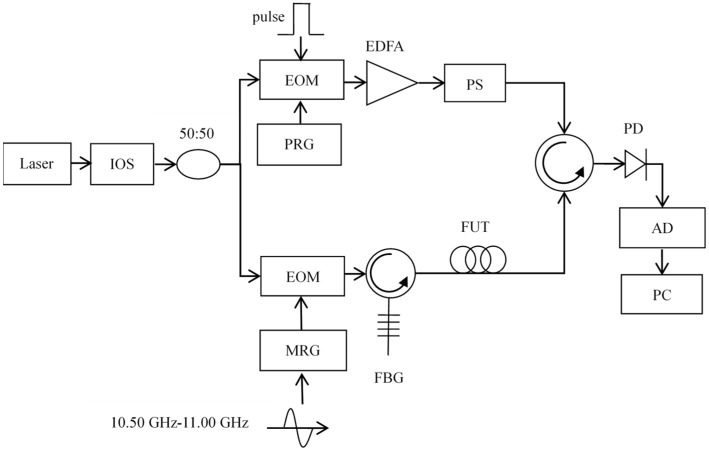
BOTDA experimental setup. ISO: Isolator, PRG: Pulse pattern generator. EOM: Electro-optic modulator PS: Polarization scrambler, PD: Photodetector, MRG: Microwave generator, FBG: Fiber grating filter, FUT: Fiber under test, AD: Analog-digital converter, EDFA: Erbium-doped fiber amplifier.

The BFS extraction by the AdaBoost Algorithm is studied experimentally. The configuration of the experiment is shown in Figure 7.

**Figure 8:**
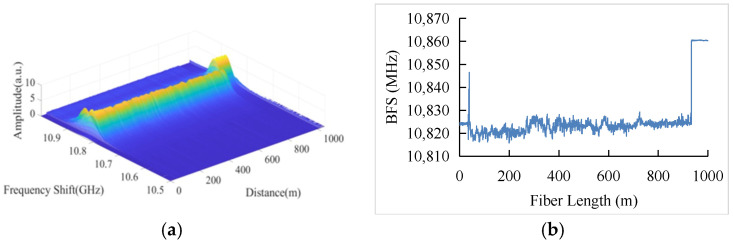
(**a**) Measured BGS distribution along 1000 km FUT with last 1000 m section heated at 30 °C, and (**b**) BFS distribution.

In the experiment, a narrow-line width laser with the wavelength of 1550 nm is used as the light source. The signal from the laser source is divided into two branches by a 50:50 coupler after passing through the optical isolator. At the upper branch, the light is modulated by an elector-optic modulator to generate pump light pulse. The power of the pump light is amplified by an Erbium-doped fiber amplifier. A polarization scrambler is introduced to suppress the polarization fluctuations. After passing through the scrambler, the pump pulse goes into the optical circulator and enters the Fiber Under Test (FUT). At the lower branch, the microwave source generates a microwave signal with the frequency of 10.500~11.000 GHz, which is modulated to the light by an electro-optic modulator. The back-scattered signal is then detected by the photodetector and converted into an electrical signal. The data are received and quantized by a 250 MSa/s acquisition card. The FUT is a 1 km single-mode optical fiber, and the last section of 80 m is heated in a thermostatic water tank, corresponding to 200 sampling points. The BGS distribution measured is shown in Figure 8. The width of the pump pulse is 25 ns, and the averaging time is 10,000. The frequency scanning step is 1 MHz.

**Figure 9:**
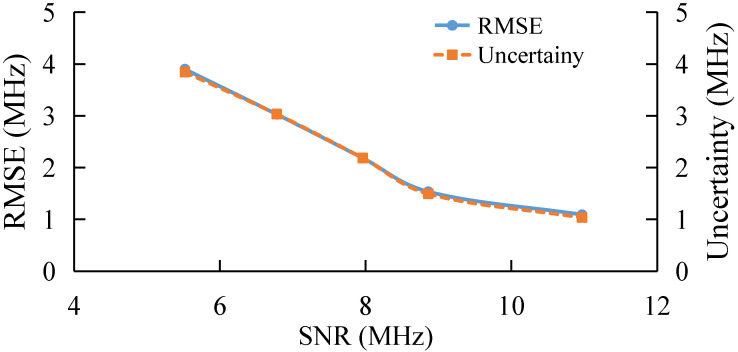
Experimental results under different SNR.

The tolerance of AdaBoost to different SNR is also studied, as shown in Figure 9, where the width of pump pulse is 20 ns and the frequency step is 1 MHz. The averaging time is selected to be 2, 4, 8, 16, and 50, separately, corresponding to the measured SNRs of 5.52 dB, 6.78 dB, 7.96 dB, 8.86 dB, and 10.96 dB, respectively. The trend of the experiment is consistent with that of the simulation. With the increase in SNR, the extraction accuracy of BGS decreases gradually.

**Figure 10:**
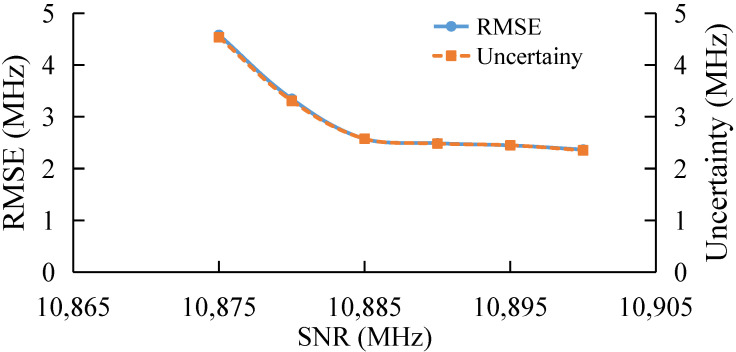
Experimental results under different cut-off frequency.

Moreover, the performance of the algorithm is also investigated under different cut-off frequency. The initial frequency is fixed at 10,800 MHz, the pump pulse is 20 ns frequency step at 1 MHz, and the average time is 4, corresponding to an SNR of 6.78 dB. The result is shown in Figure 10. The experimental result is consistent with the simulation result. The performance of the AdaBoost Algorithm becomes stable with the increase in cut-off frequency.

**Figure 11:**
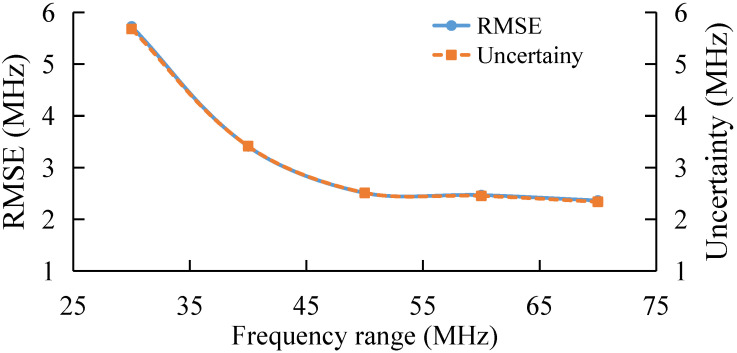
Experimental results under different frequency range.

Similarly, in order to verify the relationship between AdaBoost performance and frequency range, tests are conducted for the frequency range of 10,825–10,895 MHz. The result is shown in Figure 11. As the frequency range decreases, the BFS extraction performance of AdaBoost gradually decreases, which is consistent with the numerical simulation.

**Figure 12:**
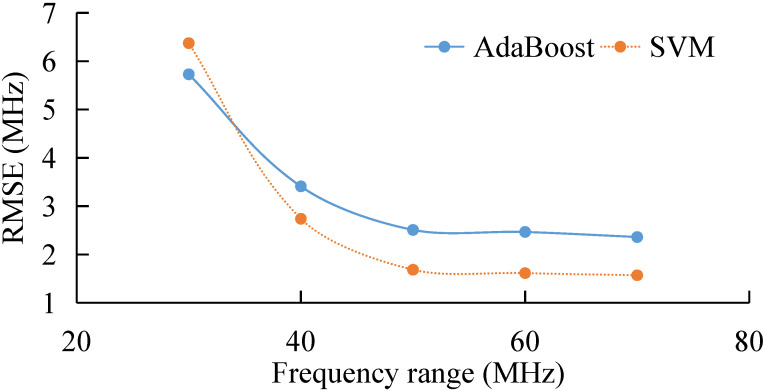
RMSE of the extracted BFS under different frequency range by AdaBoost and SVM.

Here, we compare extraction by both AdaBoost and SVM. The result is shown in a newly added figure (Figure 12). Figure 12 shows that the RMSE of the extracted BFS decreases rapidly with the increase in frequency range, due to the increase in the sampling points of BGS. RMSE of the BFS extracted by AdaBoost varies from 5.8 MHz down to 2.6 MHz, while for SVM, the RMSE varies from 6.3 MHz down to 1.5 MHz. When the frequency range is less than 35 MHz, the accuracy (RMSE) of AdaBoost is better than that of SVM. The reason may be that we use linear kernel function to train the SVM, which introduces error for small sampling sets. It is interesting to note that when the frequency range is larger than 35 MHz, the accuracy (RMSE) of SVM shows a better performance than that of AdaBoost, and the deviation between both methods is about 1 MHz. This suggests that the SVM with the linear kernel is a better choice for sampling sets which cover a large range of BGS.

**Figure 13:**
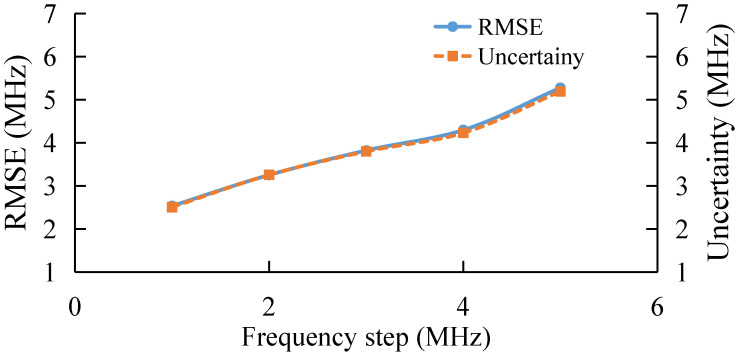
Experimental results under different frequency step.

Finally, the performance of the algorithm is also studied experimentally under different frequency step, as shown in Figure 13. With the increase in frequency step, the RMSE and uncertainty of the AdaBoost Algorithm gradually increase, which verifies the simulation conclusion that the extraction error of BFS by AdaBoost gradually increases when the sparsity of the feature set becomes larger and the number of features becomes smaller.

The simulation and experimental results are compared. Although the trend of the experiment agrees with that of the simulation, there is a deviation between the result of the simulation and that of the experiment. The deviation is about 3 MHz. This may be because the type of noise source is different, e.g., in the simulation, we use Gaussian white noise as the noise source, while in the experiment, the actual noise may include the amplified spontaneous noise (ASE) from the Erbium-doped fiber amplifier, the relative intensity noise (RIN) and phase noise from the laser source, and the thermal noise from the photo-detector.

Distributed fiber sensors can be used to measure the temperature or strain distribution along the fiber. If the fiber is bound tightly with a large-scale structure, by using distributed fiber sensors, we can obtain the total strain distribution of the bridge.

In principle, when the pump and probe enter the fiber from two ends separately, Brillouin scattering may occur, and there will be a BGS at each position along the fiber. By extracting methods, we can obtain BFS from the peak of BGS.

**Figure 14:**
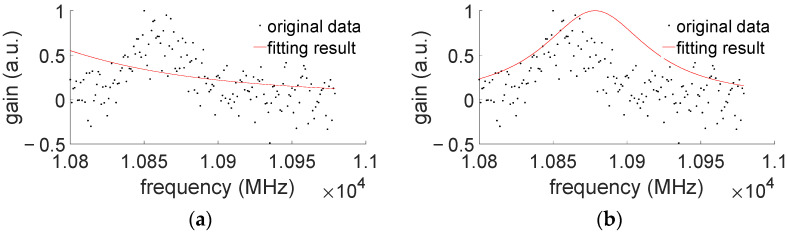
Curve fitting, (**a**) Error fitting, and (**b**) Inappropriate fitting.

Two different extracting methods are also compared. For the case when BGS has intense noise (shown in Figure 14), curve-fitting does not always lead to a good fitting result, due to possibly poor initial value settings. It is possible that the curve-fitting could result in a fitting failure, as shown in Figure 14. However, for our method of AdaBoost, we can still find BFS from a noisy BGS. Take Figure 14, for example; here, the BFS extraction by AdaBoost is about 10,860 MHz, and the extracting error is near 1 MHz. We believe this may show the advantage of the proposed AdaBoost extracting method.

## 5. Conclusions

In this study, we propose a novel method of BFS extraction using the AdaBoost Algorithm. The extracted BFS are divided into different categories, and the weak classifiers constructed by decision trees are combined to form strong classifiers. The RMSE and uncertainty of the extracted BFS can be 1 MHz with a frequency scanning step of 1 MHz and an SNR of 11 dB. The BFS extraction performance under different SNR, cut-off frequency, frequency range, and frequency step is also studied by both simulation and experiment. Results show that the BFS extraction error increases with the decrease in SNR and frequency range, and with the increase in frequency step. The trend of the experiment agrees with that of the simulation. In addition, we compare the extracting performance of both AdaBoost and SVM. Results show that when the frequency range is less than 35 MHz, the accuracy (RMSE) of AdaBoost is better than that of SVM. This may be because we use the linear kernel function to train the SVM, which introduces error for small sampling sets. The comparison between AdaBoost and curve-fitting is also presented, and the result shows that our proposed BFS extracting method is more stable than curve-fitting, especially for the case when BGS has intense noise. It is believed that AdaBoost is a good candidate for BFS extraction from weak BGS. We would like to further investigate the performance of AdaBoost and other methods, such as ANN.

## Data Availability

The data presented in this study are available on request from the corresponding author.
